# Effect of Porosity Gradient on Mechanical Properties of Cellular Nano-Composites

**DOI:** 10.3390/polym12030681

**Published:** 2020-03-19

**Authors:** Josef Jancar, Klara Zarybnicka, Jan Zidek, Frantisek Kucera

**Affiliations:** CEITEC, Brno University of Technology, Purkynova 118, 612 00 Brno, Czech Republic; klara.zarybnicka@ceitec.vutbr.cz (K.Z.); jan.zidek@ceitec.vutbr.cz (J.Z.); kuceraf@fch.vut.cz (F.K.)

**Keywords:** nano-composites, cellular materials, porosity gradient, mechanical properties

## Abstract

With their hierarchical architectures incorporating gradients in composition, porosity, and orientation, natural materials have evolved optimized balance of mechanical properties. Deciphered from the structure of bamboo, we prepared cellular solids with convex and/or concave porosity gradient and investigated their static mechanical and impact properties. Non-monotonous porosity dependences of tensile, crush, and impact strength were related to the shape of porosity gradient rather than to the properties of the wall material alone. Our results provide experimental evidence, that novel mechanically robust low density additively fabricated cellular nano-composites with convex porosity gradient satisfy the structural requirements of lightweight engineering parts. Moreover, novel functions, such as reduced flammability or electrical conductivity, can easily be introduced by selecting the type and spatial organization of nanoparticles and cellular structure of the cellular micro-particles (CMPs).

## 1. Introduction

Novel lightweight engineering materials must integrate desired combination of stiffness, strength, and toughness while enabling their high throughput fabrication into complex shapes [1]. High resistance to impact loading is among the most important properties of lightweight materials intended for structural applications in automotive parts, personal protection, and sporting goods [2,3,4,5,6,7]. To enhance fracture resistance of a cellular solid, local stiffness and strength should structurally be architected to decrease from the surface towards the interior, enabling crack deflection and reducing the crack driving force while preserving structural stiffness. This helps to suppress localization of deformation and damage development and dissipate mechanical energy towards the interior, making any cracking in the inward direction increasingly more difficult. 

Syntactic foams are low-density materials with properties qualifying them for use in demanding non-structural applications from deep-sea exploration to aerospace [8,9]. Traditional syntactic foams consist of a compact polymer mixed with stiff and brittle hollow glass microspheres (HGM) [10,11,12,13,14]. With few reported exceptions, the elastic moduli, tensile and crush strengths, critical stress intensity factor, and the strain energy release rate of syntactic foams decrease monotonously with increasing relative porosity, e.g., with increasing the HGM content [15,16,17,18,19]. 

Gradient porosity syntactic foams with enhanced static fracture and impact resistance have already been prepared by varying HGM size and volume fraction [20,21,22,23]. However, except for some metallic foams [16], no attempt has so far been reported on the replacement of rigid and fragile HGMs with cellular micro-particles (CMPs). These mechanically robust CMPs are assumed to suppress brittle failure and minimize structural instabilities. In addition, CPMs can introduce novel extrinsic deformation mechanisms, which can be tuned by changing their composition and cellular architecture. By controlling the nano-scale intrinsic and micro- to macro-scale extrinsic deformation processes, CMPs can enhance the balance of stiffness, strength, and toughness, substantially, in comparison with the traditional syntactic foams. 

In this paper, we report on preparation of cellular nano-composite micro-particles CMPs for replacing the rigid and brittle HGMs in thermoplastic syntactic foams. Static mechanical and impact properties of cellular nano-composites with porosity gradient fabricated employing the 3D printing process are compared with the predictions made using the modified Ashby–Gibson models. Non-monotonous porosity dependence of mechanical properties are ascribed to new extrinsic deformation processes occurring on the CMP level and their interaction with the intrinsic mechanisms in the matrix polymer.

## 2. Experimental

**Figure 1:**
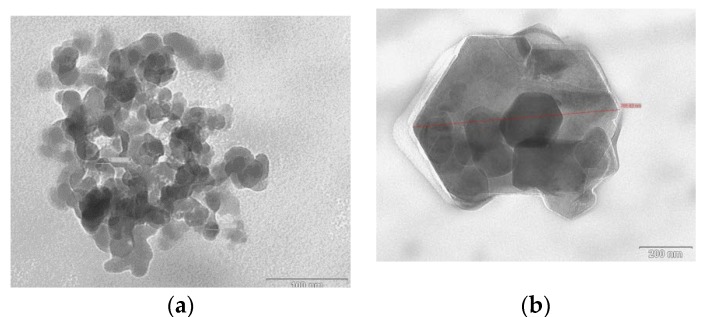
(**a**) The TEM of the prepared nanometer sized flame retardant magnesium hydroxide (nMg(OH)_2_) nanoparticles with the typical size of 20 nm. The bar in the right lower corner represents 100 nm. (**b**) The TEM of the large Mg(OH)_2_ particles. The bar in the right lower corner represents 200 nm.

Poly-methyl-methacrylate (PMMA, Evonik, Essen, Germany, ρ = 1180 kg·m^−3^, E = 3.3 GPa, G = 1.7 GPa, σ_t_ = 70 MPa and σ_c_ = 90 MPa, at 25 °C) and polypropylene impact copolymer (ICPP, Unipetrol, Prague, CZ, ρ = 960 kg·m^−3^, E = 1.7 GPa, G = 0.7 GPa, σt = 30 MPa and σc = 42 MPa, at 25 °C) were used as the matrices. The random ethylene-propylene copolymer (EPR) elastomer (Dutral 154, Buna-Werke GmbH, Schkopau, Germany) was used as the matrix for rubbery CMPs. The nanometer sized flame retardant magnesium hydroxide (nMg(OH)_2_) filler with the average particle size of 20 nm and the specific surface area of 125 m^2^/g (Figure 1) was used as prepared without any additional surface modification [24].

**Scheme 1:**
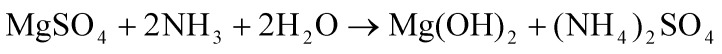
Synthesis of magnezium hydroxide nano particles.

**Figure 2:**
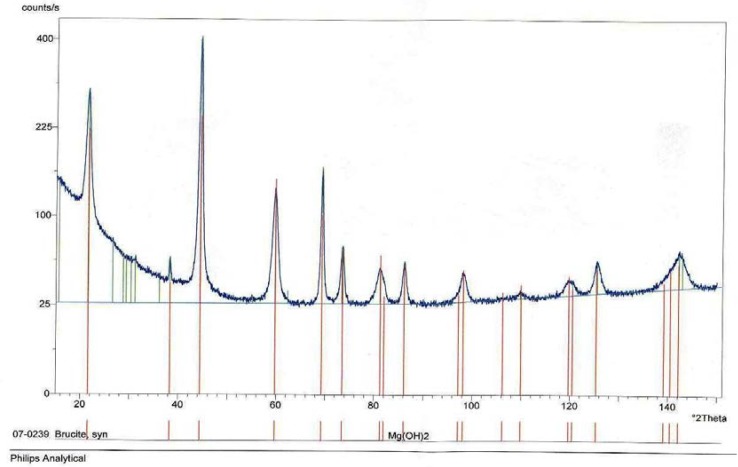
The XRD spectrum of nano Mg(OH)_2_ prepared in this work and confirming the brucite crystal structure of the precipitated nMg(OH)_2_ particles.

The platelet shaped nano-Mg(OH)_2_ was precipitated from MgSO_4_ and NH_3_ in water according to the Scheme 1:

The reaction was controlled by adjusting the pH of the solution to the pH = 10. The prepared Mg(OH)_2_ nano-particles were characterized by employing light scattering (Malvern-Zetasizer 3000 HS, Malvern Panalytical, Ltd., Enigma Business Park, Grovewood Rd, Malvern WR14, UK), Brunauer-Emmett-Teller (BET), SEM/TEM (Mira 3, Tescan, Brno, CZ), and XRD (Philips X´Pert PRO, Malvern Panalytical, Ltd., Enigma Business Park, Grovewood Rd, Malvern WR14, UK). The Mg(OH)_2_ nano-particles exhibited irregular platelet shape with the average platelet thickness of (10 ± 2) nm and width of (20 ± 7) nm (Figure 1a). Small portion (less than 10%) of particles exhibited width of (800 ± 110). The nMg(OH)_2_ specific surface area was (115 ± 25) m^2^/g and the density was (1.75 ± 0.05) g/cm^3^, and a brucite crystal structure (Figure 2).

Glassy CMPs were prepared by mixing the PMMA and nMg(OH)_2_ with methacrylate monomer containing the initiator and foaming agent employing the “dough” technique [25]. Then, the dough was foamed at 90 °C in the oven, cured in a curing device at enhanced pressure followed by milling to yield the glassy CMPs with the average particle diameter of 50 µm. The rubbery CMPs were prepared by solution blending nMg(OH)_2_, EPR elastomer, and the foaming agent. After evaporating the solvent, the EPR/nMg(OH)_2_ was foamed at 150 °C. Then, the rubbery foam was frozen in liquid nitrogen followed by milling using a ball mill into rubbery CMPs with the average particle size of 40 µm. Syntactic foams were prepared by compounding the neat polymer matrices with glassy and/or rubbery CMPs, employing a co-rotating twin-screw extruder Lab 2000 (Brabender OHG, Duisburg, Germany).

**Figure 3:**
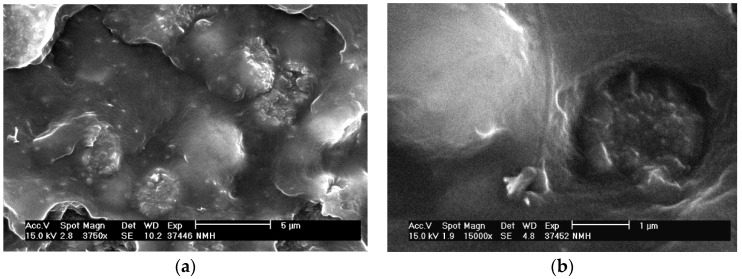
(**a**) The SEM micrograph of the notched specimens broken in a three-point bending in liquid nitrogen depicting the rubbery CMPs embedded in the ICPP matrix and revealing the porosity contained solely within the CMPs. (**b**) At larger magnification, good interfacial adhesion between the rubbery CMP and the ICPP matrix is observed.

**Figure 4:**
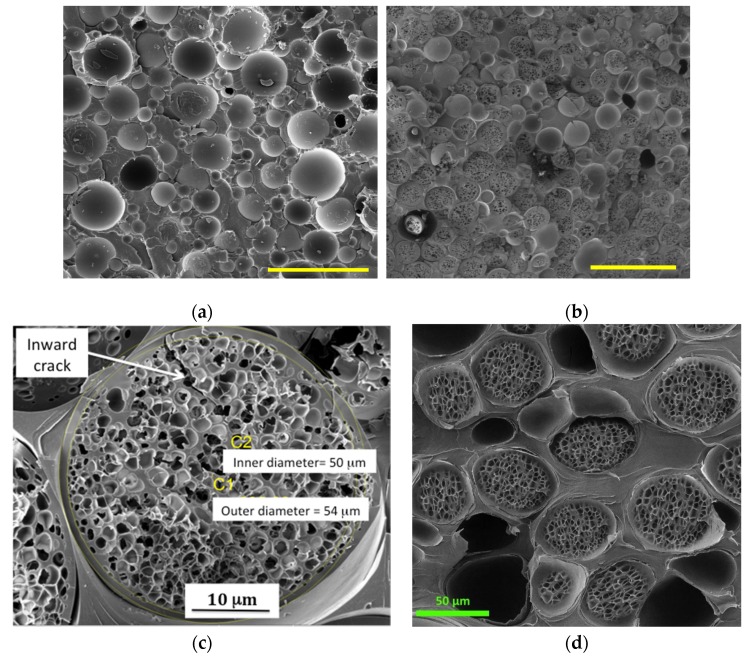
(**a**) SEM micrograph of the traditional syntactic foam consisting of the poly-methyl-methacrylate (PMMA) filled with hollow glass microspheres. (**b**) SEM micrographs of the PMMA/(PMMA/nMg(OH)_2_) syntactic foam with glassy cellular micro-particles (CMPs). (**c**) SEM micrograph of the glassy CMP in the PMMA syntactic foam broken in liquid nitrogen. In average, the CMPs consist of approximately 2 µm thick wall and cellular core, in which the pore size gradually increases inwards. An inward crack initiated in the solid shell and propagated into the cellular core where it went to an arrest is depicted in the top of the CMP. (**d**) The syntactic foam with rubbery CMPs embedded in the polypropylene impact copolymer (ICPP) matrix.

All the prepared syntactic foams contained (5.1 ± 0.2) vol% of the nMg(OH)_2_. The nMg(OH)_2_ nano-particles were preferentially contained within the CMPs (Figure 3). Most of the porosity is located inside or near the surface of the CMPs. In addition, the NPs become integrated into the cell walls within the CMPs. Typical morphology of the commercial syntactic foam and the prepared syntactic foams with glassy and/or rubbery CMPs is depicted in Figure 4.

**Figure 5:**
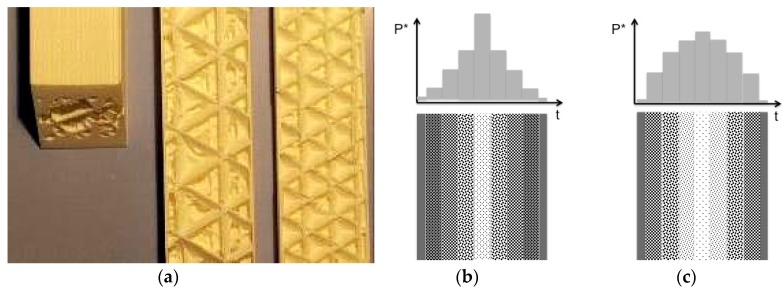
(**a**) Photograph of the convex porosity gradient specimen cross-section (left), triangular syntactic foam strut architecture in the center of the specimen (middle), and with progressively reducing pore size close to the edge of the specimen (right). The longitudinal section through the test specimens depicting the (**b**) convex and (**c**) concave porosity gradients investigated.

The desired porosity gradients were obtained by means of direct 3D printing of the novel syntactic foams employing Prusa i3 MK3S printer (Prusa Research, Prague, CZ) with the vertical resolution adjusted to 100 μm to ensure fusion of the adjacent layers. The total cross section of all beam shaped specimens was 12 by 12 mm^2^. Surface of the beams was always printed with zero porosity. The porosity gradients were fabricated employing a regularly shaped triangular arrangement of struts (Figure 5a) made of the syntactic foam with the cell size following convex (Figure 5b) and/or concave (Figure 5c) porosity profile. The relative porosity, p* = (1 − *p*_f_/*p*_m_), used as the primary structural variable, was calculated using:

(1) $$p^{*}=\frac{p_{f}}{p_{m}}=\frac{1}{p_{m}}\sum_{i=1}^{3}v_{i}p_{i}$$

where v_i_ and p_i_ is the i-th layer volume fraction and porosity, respectively.

Static tensile tests were performed employing the ElectroPuls^®^ E 10000 electro-dynamic tensile tester (Instron, Inc., 825 University Ave, Norwood, MA, USA) with optical extensometer at room temperature, 40% relative humidity, and strain rate of 10^−1^·s^−1^. Ten specimens were tested, and the average value of maximum stress and strain exhibited standard deviation of less than 18%. The Izod notched impact strength was measured employing instrumented Resil Impactor Junior (CEAST, Torino-Pianezza, Italy) at room temperature, 40% relative humidity, and the impact speed of 2.6 ms^−1^. Standard deviation of the average value determined from 10 specimens was 20%. 

Computer simulations were performed using molecular dynamics software package COMSOL^®^ (COMSOL, Inc., 100 District Avenue, Burlington, MA, USA). The simulation box had the lower edge fixed and stress of 1 MPa was applied on the top edge with free remaining side edges. The HGM was modeled using borosilicate glass cylinder (density = 2230 kg/m^3^, Young modulus = 63GPa, and Poisson ratio = 0.2) embedded in an epoxy resin (density = 1160 kg/m^3^, Young modulus = 2.75 GPa, and Poisson ratio = 0.3). The CMP was simulated as PMMA bead (density = 1190 kg/m^3^, Young modulus = 3 GPa, and Poisson ratio = 0.4) with hexagonally arranged monodisperse closed pores embedded in the same epoxy resin as in the case of the HGM. 

## 3. Results and Discussion

**Figure 6:**
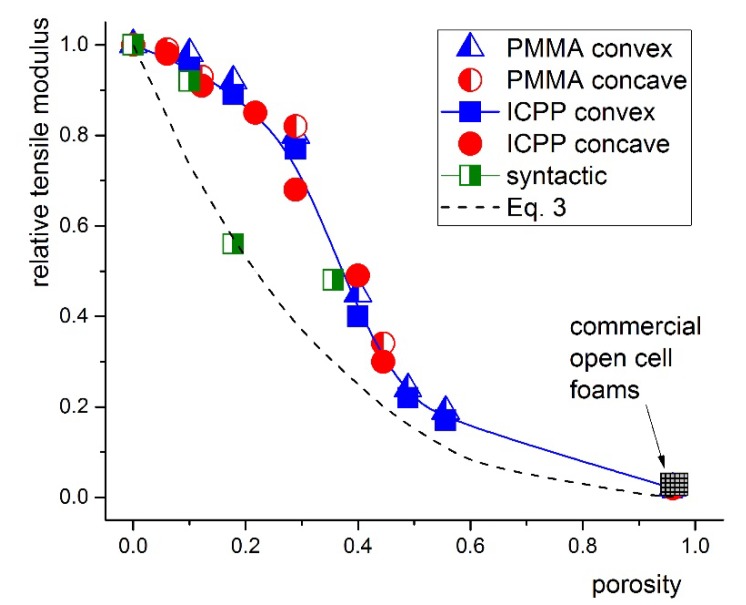
Dependence of the relative tensile modulus of the cellular composites with gradient porosity and commercial syntactic foams on the relative porosity. Dashed line represents the modified Ashby–Gibson model (Equation (3)). Solid blue line is the guide for the eyes only. Data for commercial, soft, and open cell foams are shown in the meshed box.

The tensile modulus of all the systems investigated decreased monotonously with increasing p*, regardless of the type of porosity gradient (Figure 6). Relative porosity, p* was used as the primary structural variable in the following analysis. The commercial syntactic foams complied reasonably well with Equation (2) (half-filled green squares in Figure 6). 

The porosity dependence of the tensile modulus is in a fair agreement with the prediction by the Ashby–Gibson model [26] in the form:

(2) $$\frac{E_{f}}{E_{w}}={\left(1-p*\right)}^{2}+\frac{K_{i}\left(1-v_{f}\right)}{E_{w}p*}$$

For the gradient cellular composites investigated, we replaced *p*_0_ in the original Ashby–Gibson model with the bulk modulus, *K*_i_. The *K*_i_ = 35 GPa for the HGMs and *K*_i_ = 17 GPa for the rubbery CPMs and *K*_i_ = 29 GPa for the glassy CMPs. For p* > 0.4, a fair agreement was found between the experimental data and predictions made using Equation (2). For p* < 0.4, measured stiffness exhibited positive deviation from the model predictions regardless of the type of porosity gradient.

**Figure 7:**
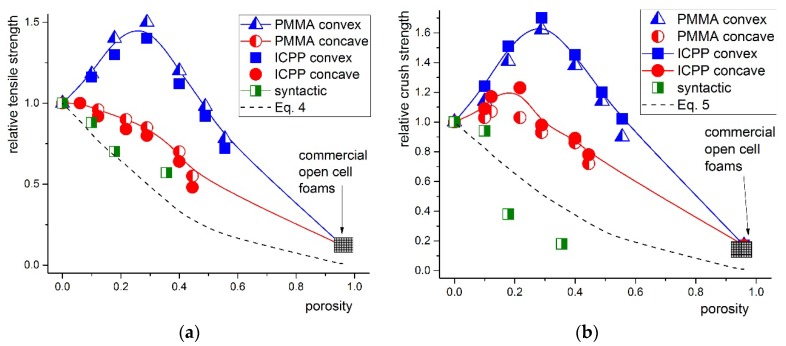
(**a**) Dependence of the tensile strength of cellular composites with gradient porosity and commercial syntactic foams on the relative porosity. (**b**) Dependence of the crush strength of gradient porosity foams and commercial syntactic foams on the relative porosity (compressive loading in the direction of density gradient). Dashed line represents the Ashby–Gibson models (Equation (3) and Equation (4)). Data for commercial soft open cell foams are shown in the meshed box.

Tensile and crush strengths of the commercial syntactic foams as well as the concave porosity gradient cellular nano-composites decreased monotonously with increasing p* (Figure 6). For the convex porosity gradient, both tensile and crush strength exhibited pronounced maximum near p* of approximately 0.4 (Figure 7).

**Figure 8:**
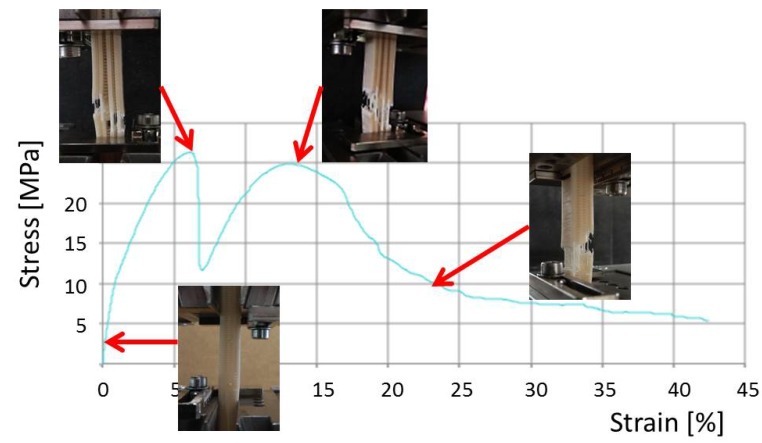
A typical tensile stress-strain curve with photographs depicting the development of damage in the cellular nano-composite beam with convex porosity gradient. The beam was cut longitudinally to visualize cell layers with different cell size.

The response of cellular nano-composites to tensile and compressive loading differed significantly, however, the trends of these dependences revealed some similarity (Figure 7). Due to the stiffness decreasing towards the center of the specimen, failure or collapse of the cellular beam occurred in multiple steps, depending on the type of loading. Beams loaded in tension failed in a sequence of events illustrated in Figure 8. In agreement with computer simulations, this process required up to five-times greater mechanical energy compared to the failure of commercial syntactic foams. Under uniaxial compression, collapse started in the central layer with the largest porosity and propagated outwards to layers with gradually decreasing porosity. The collapse of individual layers did not lead to the catastrophic failure of the cellular composite. Instead, damage propagated throughout the thickness in a stable manner.

Strength and fracture toughness have their relationship to material composition modulated by the local state of stress controlled by the structural architecture. In our case, the cell size increased from the outer surface to the middle plane in either a convex or concave manner. Under the uniaxial tension, we simply assumed the cellular composite of a parallel arrangement of layers of cells with variable cell size contributing to the overall mechanical response. Under the uniaxial compression, a serial arrangement of layers with variable porosity was assumed.

Except of the tensile strength of the commercial syntactic foams, all the experimental data deviated positively from both the simple Ashby–Gibson model for the plastic collapse strength, $\sigma_{f}^{pl}$ [26]:

(3) $$\frac{\sigma_{f}^{pl}}{\sigma_{zw}}=C_{3}\left(1-p*\right)$$

as well as from the model for the brittle crush strength, $\sigma_{f}^{cr}$:

(4) $$\frac{\sigma_{f}^{cr}}{\sigma_{w}}=C_{4}\left(1-p*\right)$$

In Equations (3) and (4), σ_yw_ and $\sigma_{w}$ are the yield strength and brittle strength of the wall material, respectively. The *C*_3_ and *C*_4_ are numerical constants. For our qualitative analysis, we have chosen both *C*_3_ and *C*_4_ equal to one.

**Figure 9:**
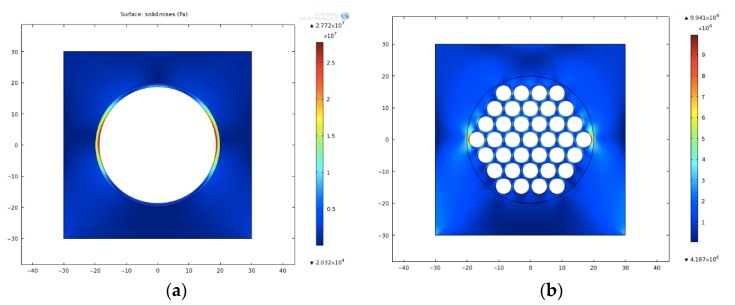
(**a**) The stress field near and in the wall of hollow glass microspheres (HGM) and (**b**) in the glassy CMP, both embedded in the glassy polymer and subjected to uniaxial compression (details in the Experimental).

As suggested by simple computer simulations, the stress field near the hollow glass bead and that near and inside the glassy CMPs differ dramatically (Figure 9). Moreover, the rubbery CMPs do not break in the brittle manner upon loading. As evidenced by the SEM observations (Figure 2c), the CPMs develop micro-cracks at their surface. However, due to the reduced stiffness towards the inside, the crack driving force is reduced and the crack is eventually stopped. Thus, the cellular inclusions contribute to the enhancement of both compressive strength and impact resistance, significantly.

Ashby and Gibson [26] proposed to express the static fracture toughness of a regular 3D cellular material in terms of the critical stress intensity factor, *K_Ic_*, as:

(5) $$\frac{K_{Ic}}{\sigma_{w}\sqrt{\pi l}}=C_{5}\left(1-p*\right)$$

**Figure 10:**
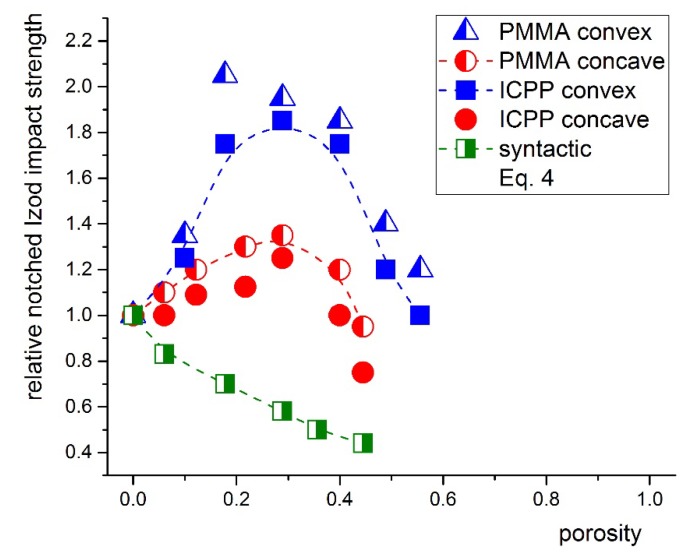
Dependence of the relative notched Izod impact strength on the relative porosity for the gradient porosity foams and commercial syntactic foams measured at room temperature and impact speed of 2.6 m/s. Dash lines serve as guides for the eye only.

In Equation (5), *l* is the initial crack length and *C*_5_ is a numerical constant. Equation (5) predicts that, for a given porosity, increasing the strength of the wall material should enhance the fracture toughness of the cellular structure. However, it also predicts a power law decrease of the *K_Ic_* with increasing the foam porosity for a given wall material. This contradicts our experimental data depicted in Figure 10. However, it qualitatively agrees with the data for the commercial syntactic foams. Hence, the Equation (5), derived for the 3D closed cell foams with spatially uniform distribution of porosity, does not provide realistic description of the gradient porosity foams investigated.

**Figure 11:**
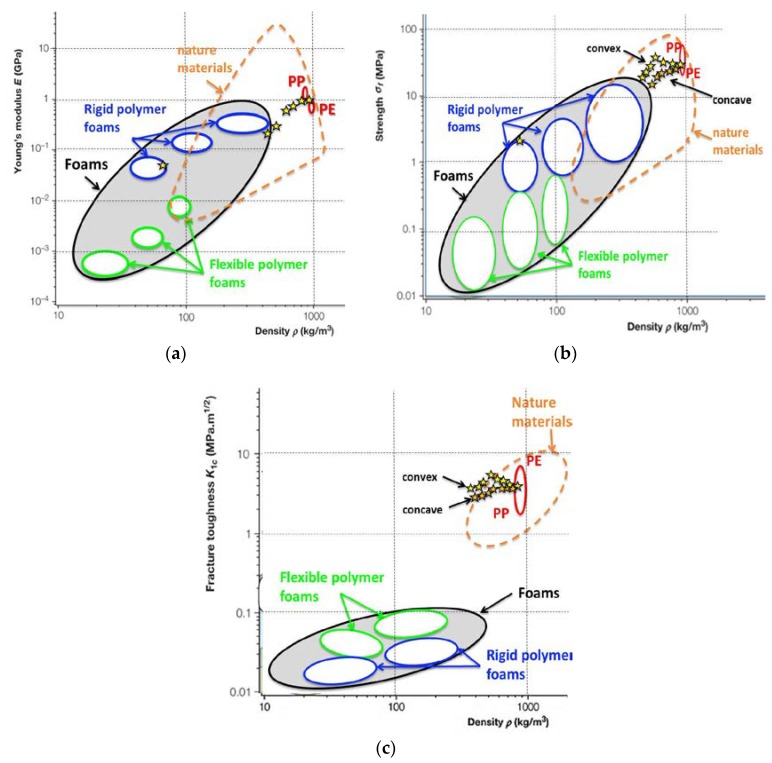
Ashby plots of the static mechanical properties versus the average density: (**a**) the Young modulus, (**b**) the tensile strength, and (**c**) the critical stress intensity factor. Stars represent experimental data obtained in this work. The Young′s modulus data for convex and concave density gradients coincided due to the logarithmic scale of the vertical axis.

Ashby plots of the elastic modulus, strength, and fracture toughness of gradient porosity cellular composites show their position within the cellular materials space (Figure 11). Properties of compact polyethylene and polypropylene and soft and rigid polymer foams are shown as references. The property space for lightweight nature materials, is demarcated by dashed line. Properties were plotted against average density, since porosity data for the reference materials were unavailable in the literature. 

Young′s modulus of the gradient porosity composites bridges the material space between the solid PE and rigid polymer foams (Figure 11a) falling within the range of natural materials. Due to the logarithmic scale of the elastic modulus axis, the data for convex and concave gradient collapsed to a single line. The tensile strength of our cellular composites matches the strongest nature materials with similar relative density with significant difference between the convex and concave porosity gradients (Figure 11b). Finally, the fracture toughness of the cellular nano-composites is two orders of magnitude greater than that for both rigid and flexible polymer foams (Figure 11c). Moreover, fracture toughness of our model gradient cellular nano-composites exceeds that for nature materials with similar density. 

Despite very low nMg(OH)_2_ content in the cellular composites, the limiting oxygen index (LOI) increased by 20% from 22.5 for the neat ICPP to 26.8 at the relative porosity of 0.4, regardless of the type of porosity gradient.

## 4. Conclusions

Novel syntactic foams with porous micro-particles, 3D printed into cellular solids with porosity gradient, exhibited non-monotonous porosity dependences of their mechanical properties. The pronounced maxima on the porosity dependences of static mechanical and impact properties, observed independent of the syntactic foam used, were ascribed to the additional extrinsic fracture processes introduced by the tough CMPs contained in the wall material. In conclusion, the effects of the wall material composition and the architecture of the porosity on the mechanical properties were found to be comparably strong. The nMg(OH)_2_ in the CMPs increased the LOI of the cellular solids by approximately 20% compared to the solid polymer. Our results suggest that cellular solids with convex porosity gradient additively fabricated using the novel syntactic foams are attractive candidates for lightweight engineering parts with enhanced impact resistance and reduced flammability.
